# Supplementary material for: Macrocycle-stabilization of its interaction with 14-3-3 increases plasma membrane localization and activity of CFTR
Source: Nat Commun. 2022 Jun 23;13:3586. doi: 10.1038/s41467-022-31206-6 (PMC9226124; doi:10.1038/s41467-022-31206-6)
Supplement: Supplementary file 3 — Reporting Summary [file 41467_2022_31206_MOESM3_ESM.pdf]

nature portfolio

Corresponding author(s): Christian Ottmann

Last updated by author(s): May 23, 2022

Reporting Summary

Nature Portfolio wishes to improve the reproducibility of the work that we publish. This form provides structure for consistency and transparency in reporting. For further information on Nature Portfolio policies, see our Editorial Policies and the Editorial Policy Checklist.

Statistics

For all statistical analyses, confirm that the following items are present in the figure legend, table legend, main text, or Methods section.

n/a	Confirmed	
☐	☒	The exact sample size (n) for each experimental group/condition, given as a discrete number and unit of measurement
☐	☒	A statement on whether measurements were taken from distinct samples or whether the same sample was measured repeatedly
☒	☐	The statistical test(s) used AND whether they are one- or two-sided<br>*Only common tests should be described solely by name; describe more complex techniques in the Methods section.*
☒	☐	A description of all covariates tested
☐	☒	A description of any assumptions or corrections, such as tests of normality and adjustment for multiple comparisons
☐	☒	A full description of the statistical parameters including central tendency (e.g. means) or other basic estimates (e.g. regression coefficient) AND variation (e.g. standard deviation) or associated estimates of uncertainty (e.g. confidence intervals)
☒	☐	For null hypothesis testing, the test statistic (e.g. F, t, r) with confidence intervals, effect sizes, degrees of freedom and P value noted<br>*Give P values as exact values whenever suitable.*
☒	☐	For Bayesian analysis, information on the choice of priors and Markov chain Monte Carlo settings
☒	☐	For hierarchical and complex designs, identification of the appropriate level for tests and full reporting of outcomes
☐	☒	Estimates of effect sizes (e.g. Cohen's d, Pearson's r), indicating how they were calculated

Our web collection on statistics for biologists contains articles on many of the points above.

Software and code

Policy information about availability of computer code

Data collection	PETRA III Synchrotron, DESY BEAMLINE P11
Data analysis	XDS (vNov 1 2016), CCP4/Scala (v7.1), Coot (v0.9.6), Phenix/Phaser (v1.17.1_3660)

For manuscripts utilizing custom algorithms or software that are central to the research but not yet described in published literature, software must be made available to editors and reviewers. We strongly encourage code deposition in a community repository (e.g. GitHub). See the Nature Portfolio guidelines for submitting code & software for further information.

Data

Policy information about availability of data

All manuscripts must include a data availability statement. This statement should provide the following information, where applicable:

- Accession codes, unique identifiers, or web links for publicly available datasets
- A description of any restrictions on data availability
- For clinical datasets or third party data, please ensure that the statement adheres to our policy

Data availability statement is present in the manuscript and contains the Data Source, Supplementary Information and PDB file locations (https://www.rcsb.org/structure/7QI1 and https://www.rcsb.org/structure/2C23).

Field-specific reporting

Please select the one below that is the best fit for your research. If you are not sure, read the appropriate sections before making your selection.

- [x] Life sciences
- [ ] Behavioural & social sciences
- [ ] Ecological, evolutionary & environmental sciences

For a reference copy of the document with all sections, see nature.com/documents/nr-reporting-summary-flat.pdf

Life sciences study design

All studies must disclose on these points even when the disclosure is negative.

Sample size	No sample-size calculation was performed.
Data exclusions	No data was excluded.
Replication	All attempts on replication were successful.
Randomization	No experimental groups were allocated.
Blinding	No blinded studies since there are no patients involved.

Reporting for specific materials, systems and methods

We require information from authors about some types of materials, experimental systems and methods used in many studies. Here, indicate whether each material, system or method listed is relevant to your study. If you are not sure if a list item applies to your research, read the appropriate section before selecting a response.

Materials & experimental systems

n/a	Involved in the study	
☐	☒	Antibodies
☐	☒	Eukaryotic cell lines
☒	☐	Palaeontology and archaeology
☒	☐	Animals and other organisms
☒	☐	Human research participants
☒	☐	Clinical data
☒	☐	Dual use research of concern

Methods

n/a	Involved in the study	
☒	☐	ChIP-seq
☒	☐	Flow cytometry
☒	☐	MRI-based neuroimaging

Antibodies

Antibodies used	Immunoprecipitation:<br>• Anti-CFTR mouse monoclonal M3A7 --> ab270238 abcam, 0.5 mg for 1 mL of beads<br>• ANTI-PAN 14-3-3 mouse monoclonal antibody (H8) --> SC1657 Santa Cruz, 0.5 mg for 1 mL of beads<br>• Anti-CFTR rabbit polyclonal --> ab181782 abcam, 1/250 dilution<br>• Anti-PAN 14-3-3 Rabbit monoclonal antibody (Y62) --> ab32560 abcam, 1/500 dilution<br>• Goat anti-rabbit HRP --> SC2004 Santa Cruz, 1/1000 dilution<br><br>CFTR trafficking assay:<br>• Mouse monoclonal anti-HA antibody (HA-7) --> H9658 Sigma, 1:150 dilution in PBS<br>• Anti-mouse IgG conjugated with FITC --> F5262 Sigma, 1:100 dilution in PBS
Validation	ab270238 abcam --> Application: IHC-P<br>SC1657 Santa Cruz --> Website: "Pan 14-3-3 Antibody (H-8) is recommended for detection of pan 14-3-3 of mouse, rat, human and avian origin by WB, IP, IF, IHC(P), FCM and ELISA; also reactive with additional species, including and equine, canine, bovine, porcine and avian" (cited in 216 publications)<br>ab181782 abcam --> Suitable for WB, ICC/IF<br>ab32560 abcam --> Suitable for: WB, IHC-P, ICC/IF<br>H9658 Sigma --> Suitable for: Immunocytochemistry, IP, ELISA, WB

Eukaryotic cell lines

Policy information about cell lines

Cell line source(s)	HEK293 (GripTiteTM cells purchased from Invitrogen and modified by the addition of stably expressing CFTR, Carlile et. al. 2015), BHK (stably expressing CFTR cells, published in Carlile et. al. in 2007) and CFBE (CFBE41o- derived from a CF patients bronchial epithelial cells and stably infected with vectors containing either wt or F508del-CFTR, Carlile et. al. 2016)
Authentication	Not authenticated
Mycoplasma contamination	Negative
Commonly misidentified lines (See ICLAC register)	No commonly misidentified cell lines were used in this study
